# OTX1 promotes TNBC cell proliferation and tumor growth through the ERK pathway

**DOI:** 10.1016/j.gendis.2025.101642

**Published:** 2025-04-11

**Authors:** Huaying Xie, Dihe Gong, Huaqiang Zhong, Jurui Luo, Liangjie Yu, Meimei Gao, Zihao Xu, Wen Yun, Yongrui Bai, Jian Sun

**Affiliations:** a Department of Radiation Oncology, Ren Ji Hospital, School of Medicine, Shanghai Jiao Tong University, Shanghai 200127, China; b Department of Breast Surgery, Ningbo Hangzhou Bay Hospital, Ningbo, Zhejiang 315336, China; c Department of Oncology, The People's Hospital of Ruijin City, Ganzhou, Jiangxi 342500, China; d Department of General Surgery, Jiangsu Cancer Hospital & Jiangsu Institute of Cancer Research & The Affiliated Cancer Hospital of Nanjing Medical University, Nanjing, Jiangsu 210000, China; e Department of Breast Surgery, Obstetrics and Gynecology Hospital, Fudan University, Shanghai 200011, China

Triple-negative breast cancer (TNBC) has a poor prognosis because of its aggressive behavior, absence of specific therapies, and high recurrence.^1^ The exact molecular mechanisms that drive the progression of TNBC are not yet fully understood, thus highlighting the urgent need for discovering novel potential treatment targets. With the advancement of high-throughput technologies, the identification of dysregulated genes and pathways in TNBC has become feasible. Therefore, it is integral to identify novel therapeutic targets and improve patient outcomes.

We initially analyzed a GEO dataset comprising 165 TNBC samples and 33 paired normal breast tissues (GSE76250). We identified the differentially expressed genes between TNBC samples and their adjacent non-cancerous tissues (*n* = 33) and selected 100 of these genes to create a heatmap (Fig. S1A). Figure S1B presents the distribution of differentially expressed genes using a volcano plot. Subsequently, we conducted Gene Ontology (GO) term enrichment and Kyoto Encyclopedia of Genes and Genomes (KEGG) pathway analyses with these differentially expressed genes (Fig. S1C, D). Among these genes, orthodenticle homeobox 1 (OTX1) was markedly elevated in TNBC samples compared with the non-cancerous adjacent tissues (Fig. 1A; Fig. S1E). This result was supported by analyses from the UALCAN database, which revealed that OTX1 expression was much higher in breast cancer tissues, especially in TNBC samples (Fig. S1F, G). Similar results were also found in TNBC cell lines when compared with normal breast epithelial cells (Fig. S1H). OTX1, belonging to a member of the homeobox gene family, is widely expressed and has been observed to regulate the development of some cancer types.^2^^,^^3^ However, the regulatory effects of OTX1 on different types of cancers are inconsistent, and its role in TNBC remains unclear, emphasizing the need for further investigation to determine its molecular function in this context.

**Figure 1:**
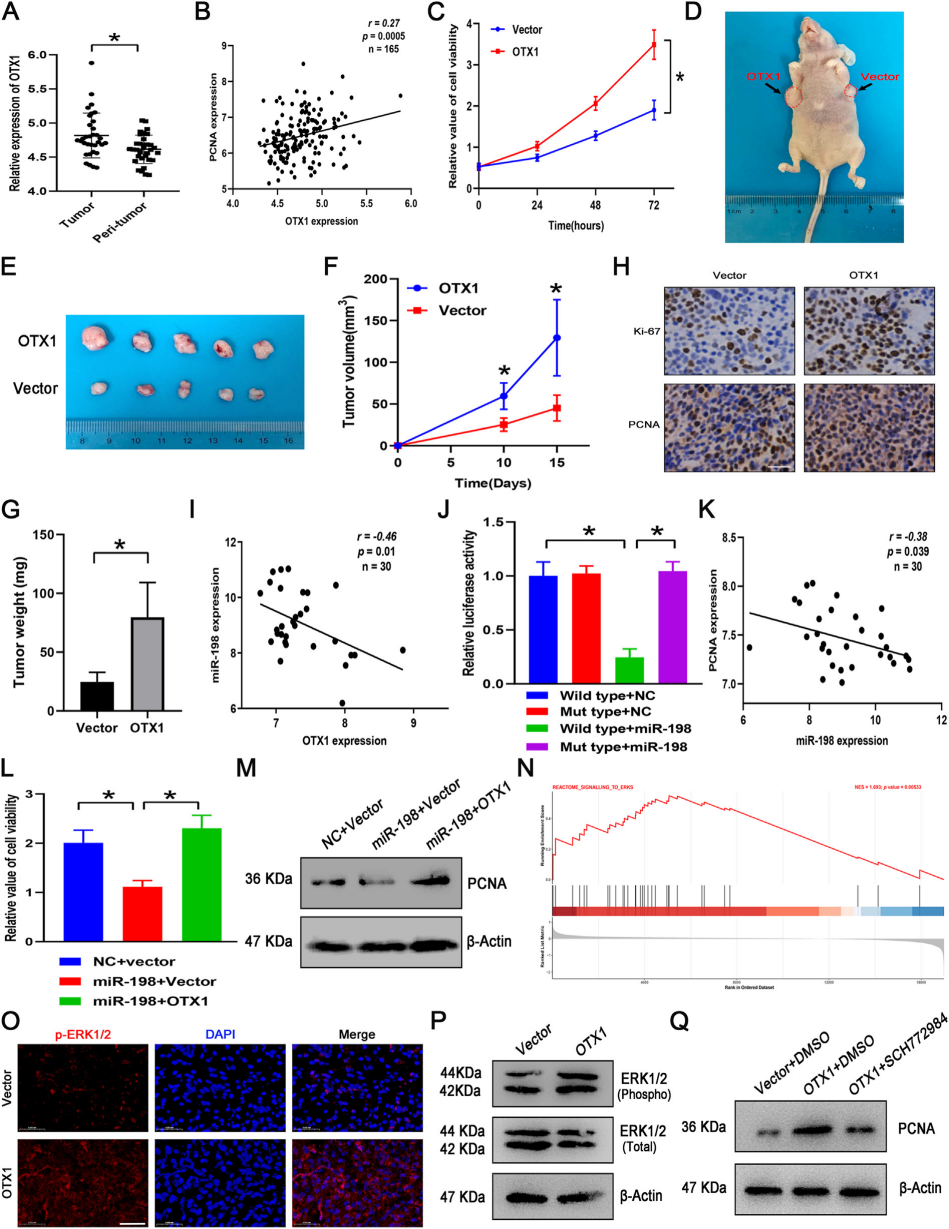
OTX1 negatively regulated by miR-198 in triple-negative breast cancer (TNBC) facilitates cell proliferation via the ERK pathway in TNBC. **(A)** OTX1 expression was markedly higher in TNBC samples. **(B)** OTX1 expression in TNBC positively correlated with PCNA expression. **(C)** OTX1 overexpression significantly enhanced cell viability in MDA-MB-231 cells. **(D, E)** Overexpression of OTX1 significantly promoted tumor growth (D), along with representative images of the tumor tissues (E) isolated from nude mice (*n* = 5). **(F, G)** Tumor volumes (F) and tumor weight (G) were significantly increased by OTX1 overexpression. **(H)** OTX1 overexpression led to a higher proportion of cells positive for Ki-67 and PCNA. Scale bar = 50 μm. **(I)** miR-198 expression showed an inverse correlation with OTX1 levels in the same TNBC tissues. **(J)** Treatment with miR-198 suppressed luciferase reporter activity in wild-type (WT) constructs, while mutant (MT) constructs remained unaffected. **(K)** Inverse association between miR-198 and PCNA expression within the same TNBC specimens. **(L)** Reduced cell viability following miR-198 treatment was reverted by restoring OTX1 expression. **(M)** OTX1 restoration alleviated the miR-198-induced reduction in the protein levels of PCNA. **(N)** Gene Set Enrichment Analysis (GSEA) revealed a positive correlation between genes implicated in the ERK pathway and heightened OTX1 expression in TNBC (REACTOME_SIGNALLING_TO_ERKS; normalized enrichment score (NES) = 1.693; *P* = 0.00533). **(O)** Immunofluorescence analysis showed that the expression of p-ERK1/2 was significantly higher in tumor sections from the OTX1 overexpression group compared with the control vector groups. Scale bar = 50 μm. **(P)** The overexpression of OTX1 promoted the phosphorylation of ERK1/2 in MDA-MB-231 cells without significantly impacting total ERK1/2 levels. **(Q)** Up-regulation of PCNA expression induced by OTX1 was mitigated by the suppression of the ERK pathway.

Upon further examining the expression of OTX1 in TNBC tissues (*n* = 165) relative to various clinicopathological variables, we observed that OTX1 was markedly elevated in patients under 50 years and those with high tumor grades (Fig. S2A, B). Additionally, TNBC patients with ki67 > 30% displayed higher OTX1 expression than those with ki67 ≤ 30% (Fig. S2C). However, no significant variation in OTX1 expression emerged between menopausal and pre-menopausal groups (Fig. S2D). To clarify whether OTX1 was linked to TNBC progression, we explored its relationship with cell proliferation and cell cycle markers. Our data revealed a positive correlation between OTX1 expression and both proliferating cell nuclear antigen (PCNA) (*r* = 0.27, *P* = 0.0005) and marker of proliferation Ki-67 (MKI67) (*r* = 0.31, *P* < 0.0001) (Fig.1B; Fig. S2E), as well as cell cycle regulators cyclin A2 (*r* = 0.29, *P* = 0.0001), CCNB2 (*r* = 0.34, *P* < 0.0001), cyclin D1 (*r* = 0.23, *P* = 0.0025), and cyclin E1 (*r* = 0.32, *P* < 0.0001) (Fig. S2F–I). Nevertheless, we surprisingly found that OTX1 expression alone was not predictive of TNBC prognosis in terms of overall survival (Fig. S2J), recurrence-free survival (Fig. S2K), or distant metastasis-free survival (Fig. S2L).

We then overexpressed OTX1 in MDA-MB-231 cells to investigate the functional roles of OTX1 in the proliferation of TNBC cells (Fig. S3A). We found that OTX1 overexpression increased cell viability, facilitated BrdU incorporation, and elevated PCNA protein levels (Fig. 1C; Fig. S3B, C). *In vivo* experiments showed that the overexpression of OTX1 promoted tumor growth and increased tumor volumes (Fig. 1D–F). This is further corroborated by observations of elevated tumor weight in instances of OTX1 up-regulation (Fig. 1G). Immunohistochemical analyses suggested that OTX1 overexpression led to a higher proportion of cells positive for Ki-67 and PCNA, indicating increased cellular proliferation *in vivo* (Fig. 1H). Additionally, the knockdown of OTX1 was also performed to evaluate its biological function (Fig. S3D). We found that OTX1 knockdown led to a decrease in cell viability, BrdU incorporation, and PCNA expression (Fig. S3E–G). Consistent effects were obtained in another TNBC cell line (MDA-MB-157; Fig. S3H–K).

It is well established that microRNAs are crucial regulators of target gene expression. To further explore the upstream regulation of OTX1, we identified which microRNAs may be responsible for its increased expression in TNBC. Using bioinformatic tools such as TargetScan and miRDiB, we predicted potential miRNAs that could target OTX1. In combination with the expression levels of miRNAs and OTX1 in TNBC tissues, we found that miR-198, which exhibited a negative correlation with OTX1 expression in the same TNBC tissue samples (*r* = −0.46; *P* = 0.01), was a potential regulator (Fig. S4A; Fig. 1I). The prospective binding sites of miR-198 was within the 3′UTR region of OTX1 and displayed a mutated UTR construct used for a luciferase reporter assay (Fig. S4B). Our findings show that treatment with miR-198 reduced luciferase activity in the wild-type group, whereas it had no impact on the mutant group (Fig. 1J). Additionally, both mRNA and protein levels of OTX1 decreased upon miR-198 exposure (Fig. S4C, D), whereas inhibition of miR-198 enhanced OTX1 expression (Fig. S4E, F). As demonstrated in Figure 1K, there was a negative correlation between the expression of miR-198 and PCNA in the same TNBC tissues. Consistently, miR-198 treatment resulted in a decrease in cell viability and BrdU incorporation, which was reversible upon restoring OTX1 expression (Fig. 1L; Fig. S5A). The miR-198-mediated down-regulation of PCNA expression was mitigated by the reintroduction of OTX1 (Fig. 1M). In contrast, suppressing miR-198 stimulated cell growth, BrdU incorporation, and elevated PCNA expression, and these effects were diminished when OTX1 was knocked down (Fig. S5B–D).

We further explore the downstream regulatory mechanisms exerted by OTX1. TNBC tissues were categorized into two groups based on the intrinsic expression levels of OTX1 (low and high expression). Gene Set Enrichment Analysis (GSEA) revealed a positive correlation in genes for the activation of the extracellular signal-regulated kinase (ERK) pathway in high expression of OTX1 compared with low expression of OTX1 (Fig. 1N; Fig. S6A). We thus determined whether the regulatory effects of OTX1 on cell proliferation were mediated by the ERK pathway in TNBC. As depicted in Figure 1O, immunofluorescence results indicated a significant increase in p-ERK1/2 expression in the tumor tissues of the OTX1 overexpression group. Consistently, OTX1 overexpression resulted in augmented phosphorylation of ERK1/2 in MDA-MB-231 cells, while the total expression of ERK1/2 was not obviously affected by the overexpression of OTX1 (Fig. 1P). Moreover, the increase of cell viability and BrdU incorporation induced by OTX1 overexpression were attenuated by an ERK pathway inhibitor (SCH772984, 1 μM) (Fig. S6B, C). OTX1 overexpression-induced PCNA expression was mitigated by the inhibition of the ERK pathway (Fig. 1Q). These findings suggest that OTX1-mediated cell proliferation in TNBC involves the ERK pathway.

In summary, our study offers new insights into the oncogenic role of OTX1 in TNBC, which is negatively regulated by miR-198. OTX1 promotes cell proliferation through the ERK pathway. These findings advance our understanding of OTX1's functions and mechanisms underlying TNBC progression, potentially offering new therapeutic approaches for this aggressive breast cancer type.

## CRediT authorship contribution statement

**Huaying Xie:** Writing – original draft, Methodology, Formal analysis, Data curation, Conceptualization. **Dihe Gong:** Writing – review & editing, Visualization, Validation, Funding acquisition, Data curation. **Huaqiang Zhong:** Writing – review & editing, Visualization, Validation, Data curation. **Jurui Luo:** Writing – review & editing, Methodology, Funding acquisition. **Liangjie Yu:** Visualization, Software. **Meimei Gao:** Writing – review & editing, Data curation. **Zihao Xu:** Writing – review & editing, Software. **Wen Yun:** Writing – review & editing, Visualization, Validation, Methodology, Investigation. **Yongrui Bai:** Writing – review & editing, Supervision, Project administration, Methodology, Conceptualization. **Jian Sun:** Writing – review & editing, Writing – original draft, Supervision, Project administration, Investigation, Funding acquisition, Conceptualization.

## Ethics declaration

All experiments involving animals were approved by the Ethics Committee of Renji Hospital, Shanghai Jiao Tong University School of Medicine.

## Funding

This study was supported by the National Natural Science Foundation of China (No. 82072923, 82102829) and Zhejiang Provincial Medical Science and Technology Program (China) (No. 2020KY903).

## Conflict of interests

The authors declared no competing interests.
