# Supplementary material for: OTX1 promotes TNBC cell proliferation and tumor growth through the ERK pathway
Source: Genes Dis. 2025 Apr 11;13(1):101642. doi: 10.1016/j.gendis.2025.101642 (PMC12466130; doi:10.1016/j.gendis.2025.101642)
Supplement: Multimedia component 1 [file mmc1.docx]

**Supplementary Methods**

**Cell Culture and Treatment**

Human TNBC cell lines (MDA-MB-231 and MDA-MB-157) were obtained from the cell bank of the Chinese Academy of Sciences (Shanghai, China). Cells were cultured in DMEM with 10% FBS and 1% penicillin-streptomycin at 37°C, 5% CO2. At 80% confluence, cells were passaged using 0.25% trypsin-EDTA for experiments. For OTX1 overexpression studies, cells were infected with the lentiviruses containing the cDNA encoding OTX1 (Genechem, Shanghai, China) or empty vector as control according to the protocols provided by the manufacturer. OTX1 knockdown was achieved by infecting cells with the lentiviruses containing OTX1 shRNA (Genechem, Shanghai, China) or a scrambled shRNA as a negative control. Real-time PCR was used for verifying the overexpression and knockdown efficiencies.

**CCK-8 Assay**

To assess cell viability, we used the Cell Counting Kit-8 (CCK-8, Dojindo Molecular Technologies). Cells were seeded at 3x10^3^ cells per well in a 96-well plate and allowed to attach for 24 hours. Subsequently, 10 µL of CCK-8 solution was added to each well with 100 µL of culture medium. After an additional 1 hour incubation at 37°C, the absorbance at 450 nm was measured using a microplate reader.

**BrdU incorporation assay**

To quantify cell proliferation, BrdU incorporation was measured using the Cell Proliferation ELISA, BrdU (colorimetric) kit from Roche Applied Science. Briefly, cells were seeded at 1x10^4^ cells per well in a 96-well plate and allowed to adhere overnight. Following the manufacturer's instructions, cells were further incubated for 24 hours after adding BrdU labeling solution. Subsequently, the cells were fixed, and anti-BrdU antibody conjugated to peroxidase was used for detection. Following a 1.5-hour incubation at 25°C, the reaction was developed with a substrate solution, and the optical density was read at 370 nm using a microplate reader.

**Western Blot**

Equal amounts of protein (30 µg) were separated by SDS-PAGE and subsequently transferred to polyvinylidene fluoride (PVDF) membranes. After blocking with 5% non-fat milk in TBST for 1 hour at room temperature, membranes were then incubated with primary antibodies overnight at 4°C. After washing with TBST, HRP-conjugated secondary antibodies were applied. Then the membranes were treated with enhanced chemiluminescence (ECL) solution (Thermo Scientific). The bands on the membranes were quantified using ImageJ software.

**Real-time PCR**

TRIzol reagent (Invitrogen) was utilized for extracting total RNA. Then RNA was reverse transcribed into cDNA with 1 µg of total RNA using the High-Capacity cDNA Reverse Transcription Kit (Applied Biosystems). Real-time PCR was conducted with SYBR Green PCR Master Mix (Applied Biosystems) on a 7500 Fast Real-time PCR system following the manufacturer manufacturer's protocol. Primers for OTX1 were designed as follows: OTX1 forward, 5’−GCCCATGCACTCACATCAC−3’ and reverse, 5’−CAGGCTCCTTGTAATCCAAGC−3’. Gene expression was normalized to beta-actin using the 2^^-ΔΔCT^ method for relative quantification.

**Luciferase Reporter Assay**

Luciferase reporter assays were performed with pGL4 luciferase reporter plasmids (Promega) containing the WT 3'-UTR region or Mutant UTR of OTX1. Briefly, cells were co-transfected with the reporter plasmid, Renilla luciferase control vector (Promega), NC, or miR-198 using Lipofectamine 3000 (Invitrogen) for the respective experimental groups. After 48 hours, cells were lysed, and luciferase activity was measured with a Dual-Luciferase Reporter Assay System (Promega) following the provided instructions. Firefly luciferase activity was normalized to Renilla luciferase activity in each sample.

**Nude mouse xenograft model**

The female BALB/c nude mice (6-8 weeks) from SLAC Laboratory Animal Co., Ltd. (Shanghai, China) were used to establish xenograft models. Animal experiments adhered to guidelines approved by the Ethics Committee of Renji Hospital, Shanghai Jiao Tong University School of Medicine. Mice were housed in SPF conditions (5 per cage, 12h light/dark cycle, food and water ad libitum). MDA-MB-231 cells (5×10^6^) overexpressing OTX1 or vector control were subcutaneously injected into bilateral flanks of nude mice, respectively. After 15 days, mice were anesthetized and sacrificed. All efforts were made to minimize animal suffering. Subsequently, tumor weights and sizes were measured. Tumor tissues underwent immunohistochemical staining following standard protocols.

**Immunohistochemistry and Immunofluorescence**

Tissue samples were fixed in 4% paraformaldehyde for 24 hours and then embedded in paraffin. Sections were cut with a microtome and mounted on poly-L-lysine-coated slides. After deparaffinization and rehydration, antigen retrieval was conducted. Sections were permeabilized with 0.3% Triton X-100 for 15 minutes for immunofluorescence. Sections were then blocked with 10% normal goat serum for 1 hour at room temperature. Primary antibodies against PCNA (1:500 dilution, Cell signaling technology, 13110), Ki-67 (1:600 dilution, Cell signaling technology, 9449) or p-ERK1/2 (1:200 dilution, Cell signaling technology, 4370) were applied and incubated overnight at 4°C. After washing with PBS, sections were incubated with biotinylated secondary antibodies and then with a streptavidin-HRP complex for immunohistochemistry, or with fluorophore-conjugated secondary antibodies in the dark for immunofluorescence, at room temperature for 1 hour. For immunohistochemistry, immunoreactivity was visualized using DAB chromogen, and sections were counterstained with hematoxylin. Images were captured with a light microscope equipped with a digital camera. For immunofluorescence, nuclei were counterstained with DAPI (1 μg/mL) for 10 minutes, and images were acquired using a fluorescence microscope equipped with a digital camera.

**Statistical analysis**

GraphPad Prism (v.8.0.1) were utilized to conduct the statistical analysis. Data were derived from a minimum of three independent experiments and are presented as mean ± standard error of the mean (SEM). Statistical significance was determined using Student's t-test or one-way ANOVA followed by Dunnett's test as appropriate. Correlations were assessed using the Pearson r coefficient. A *p*-value of 0.05 or less indicated statistical significance. The Gene Expression Omnibus (GEO) datasets used in this study are GSE76250 and GSE59595.

**Supplementary Figures**
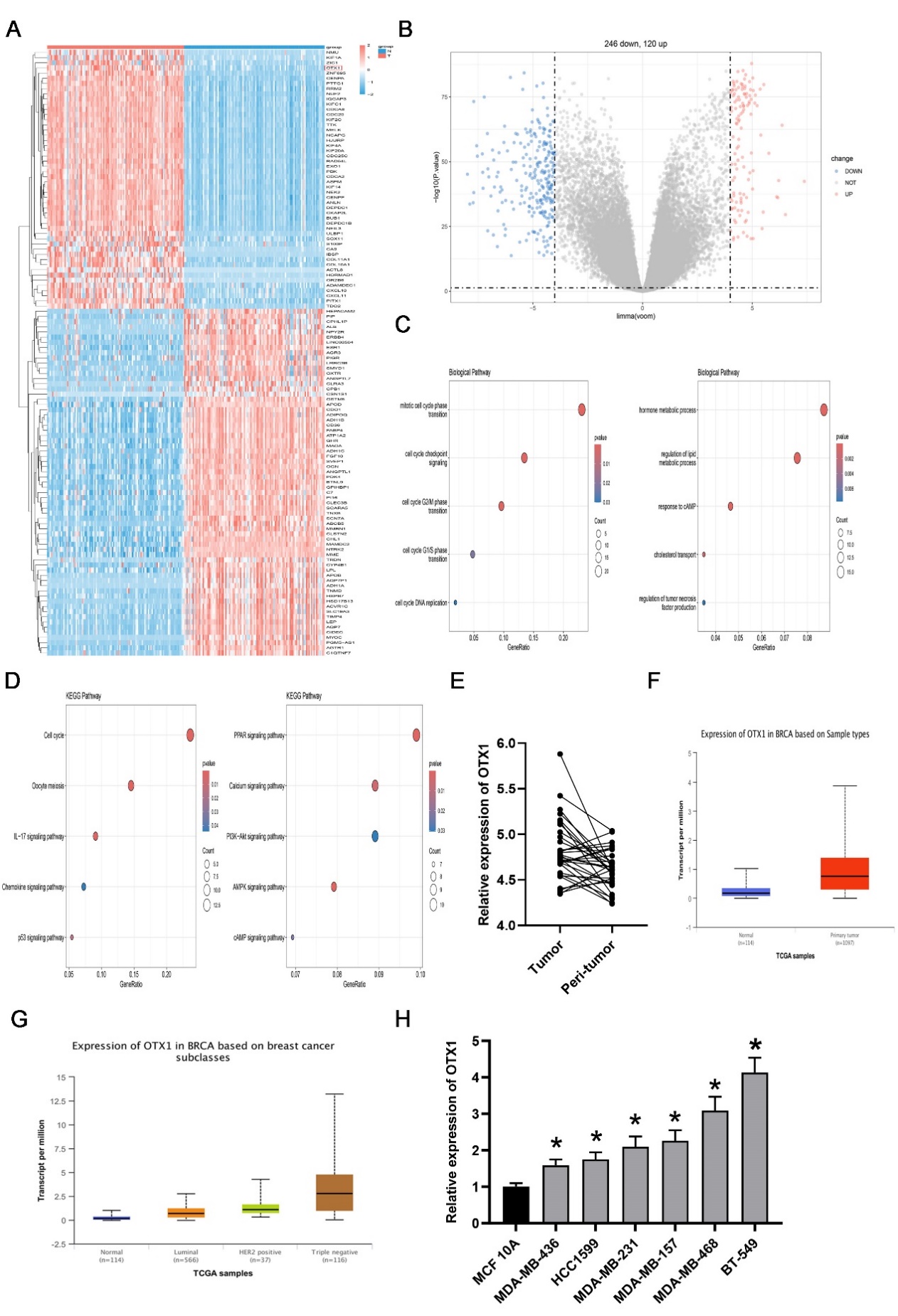


**Figure S1**

OTX1 expression is significantly upregulated in TNBC. A: A heatmap was generated from 100 genes (50 with higher expression and 50 with lower expression) showing differential expression between TNBC samples and adjacent non-cancerous tissues (n=33). B: Differentially expressed genes are depicted in a volcano plot. C and D: Gene Ontology (GO) analysis (C) and Kyoto Encyclopedia of Genes and Genomes (KEGG) pathway analysis (D) were conducted on up-regulated and down-regulated genes, respectively. E: OTX1 expression was markedly higher in TNBC samples and predominantly elevated compared to corresponding adjacent normal tissues. F: OTX1 expression was significantly elevated in breast cancer tissues. G: OTX1 expression was significantly elevated in breast cancer tissues, particularly in TNBC samples. H: The endogenous expression of OTX1 in normal breast epithelial cells (MCF 10A) and six TNBC cell lines was examined by real-time PCR. The results showed that OTX1 expression is highly expressed in TNBC cells compared with normal breast epithelial cells. * indicates *p*<0.05 compared with MCF 10A cells.


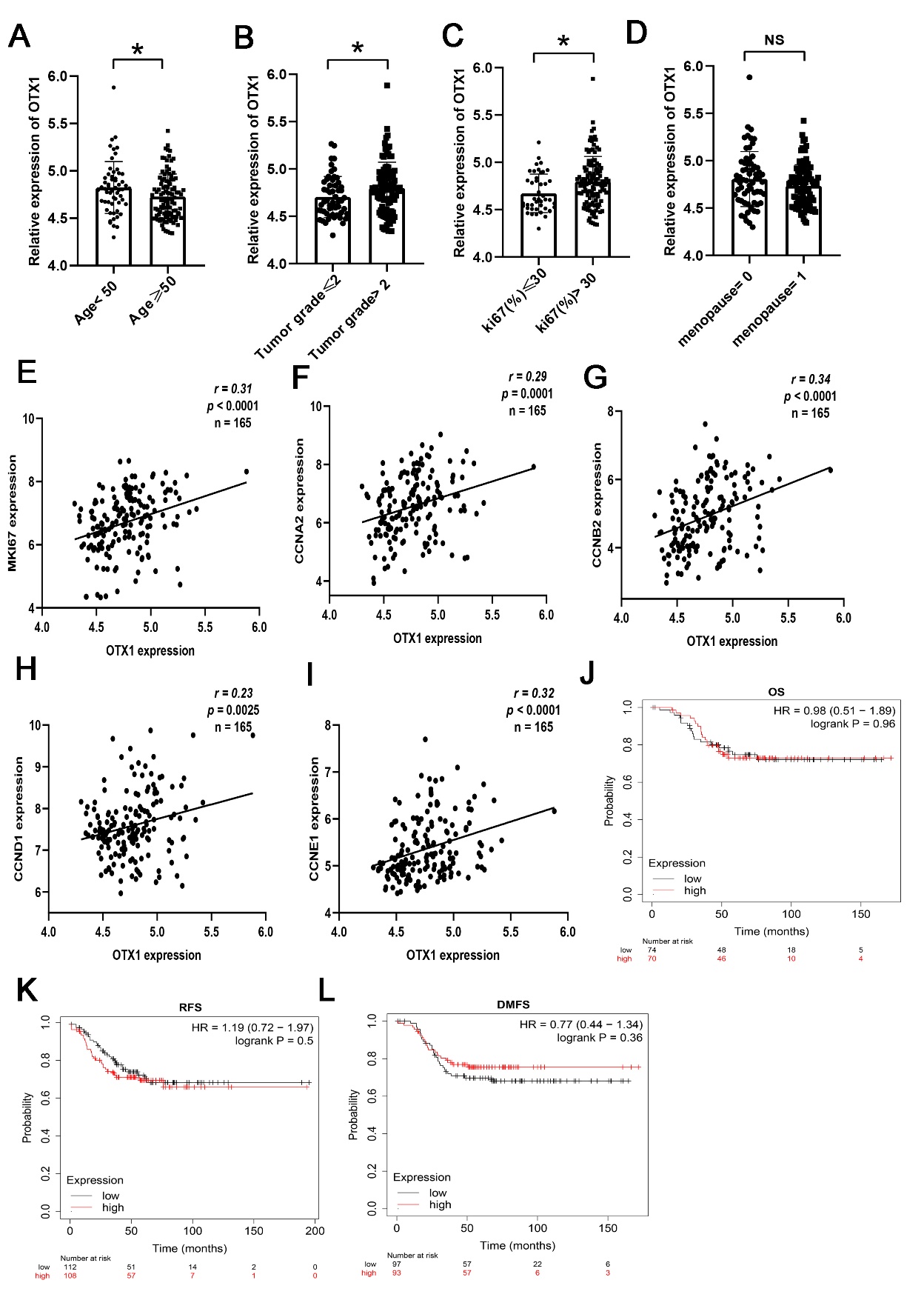


**Figure S2**

OTX1 Expression Correlates Positively with Cell Proliferation and Cell Cycle Marker Expression. A and B: Increased OTX1 expression was observed in patients under 50 years old (A) and those with higher tumor grades (B). C: TNBC patients with Ki67 > 30% exhibited higher OTX1 expression compared to those with Ki67 ≤ 30%. D: No significant differences in OTX1 expression were found between menopausal and pre-menopausal groups. E: OTX1 expression in TNBC positively correlated with MKI67 expression. F-I: OTX1 expression showed a positive correlation with cell cycle regulators CCNA2 (F), CCNB2 (G), CCND1 (H), and CCNE1 (I) in TNBC. J-L: There were no significant differences in overall survival (OS, J), recurrence-free survival (RFS, K), or distant metastasis-free survival (DMFS, L) between groups with high and low OTX1 expression in TNBC.


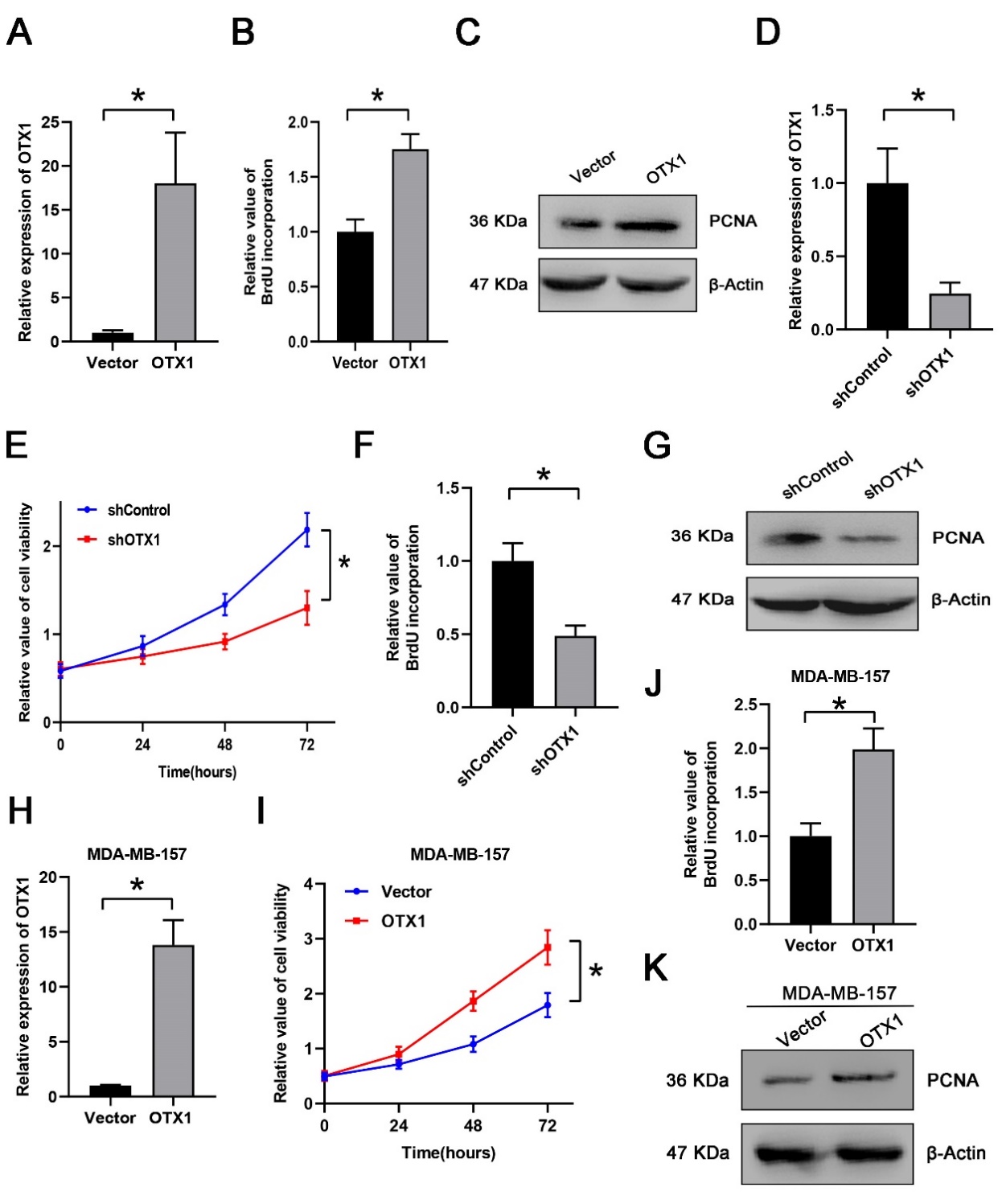


**Figure S3**

OTX1 positively regulates cell proliferation in TNBC cells. A: Overexpression efficiency was confirmed using real-time PCR in MDA-MB-231 cells. B and C: Overexpression of OTX1 increased BrdU incorporation during DNA synthesis (B) and elevated PCNA protein levels (C). D: Knockdown efficiency was confirmed via real-time PCR. E: OTX1 knockdown reduced cell viability. F and G: OTX1 knockdown inhibited BrdU incorporation during DNA synthesis (F) and decreased PCNA expression (G). H: Real-time PCR was utilized to determine the overexpression efficiency in MDA-MB-157 cells. I: Cell viability was increased by OTX1 overexpression. J and K: Overexpression of OTX1 promoted BrdU incorporation (J) and induced PCNA expression (K) in MDA-MB-157 cells.


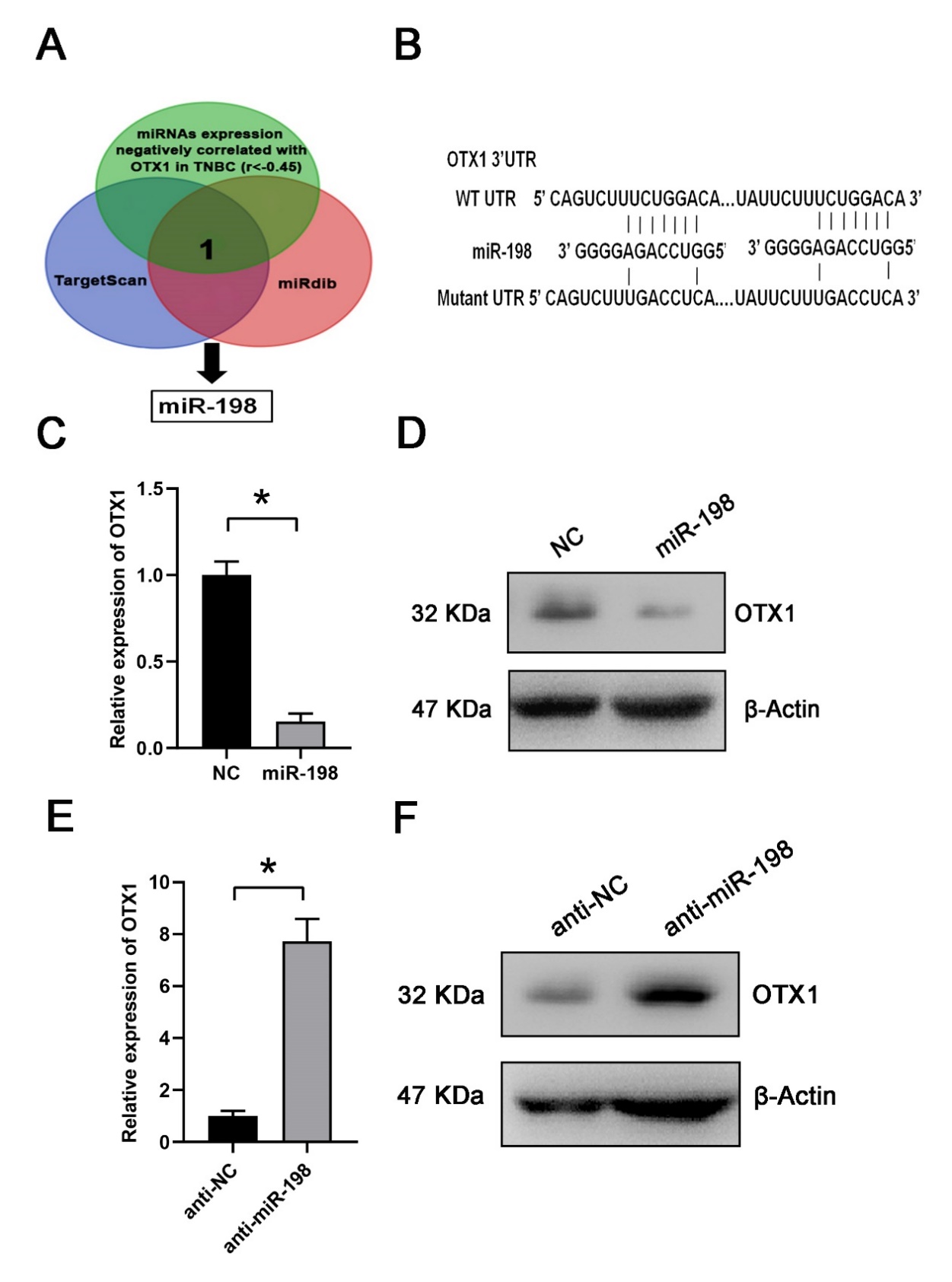


**Figure S4**

miR-198 acts as an upstream regulator of OTX1 in TNBC. A: miR-198 was identified as a conceivable regulatory element of OTX1 in TNBC tissues. B: Potential miR-198 binding sequences within the 3′UTR of the OTX1 gene are depicted. C and D: Treatment with miR-198 led to a notable suppression of both OTX1 mRNA (C) and protein (D) levels in TNBC cells. E and F: An increase in OTX1 mRNA (E) and protein (F) expression is observed following miR-198 inhibition.


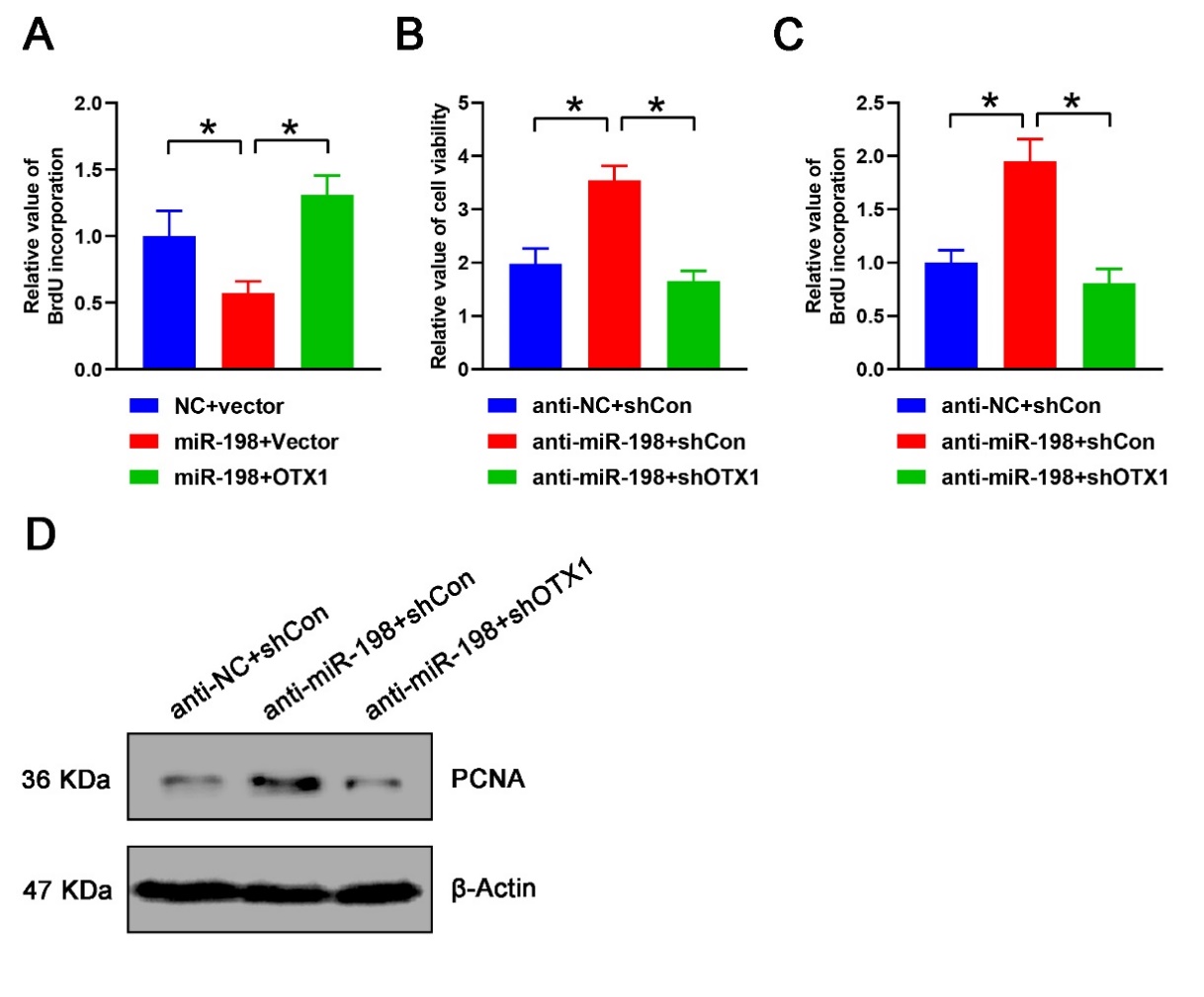


**Figure S5**

The inhibitory effects of miR-198 on cell proliferation are mediated by OTX1. A: Reduced BrdU incorporation following miR-198 treatment was reverted by restoring OTX1 expression. B: Increased cell viability due to miR-198 suppression is counteracted by OTX1 silencing. C and D: Enhancing BrdU incorporation (C) and PCNA levels (D) induced by miR-198 inhibition are attenuated by OTX1 depletion.


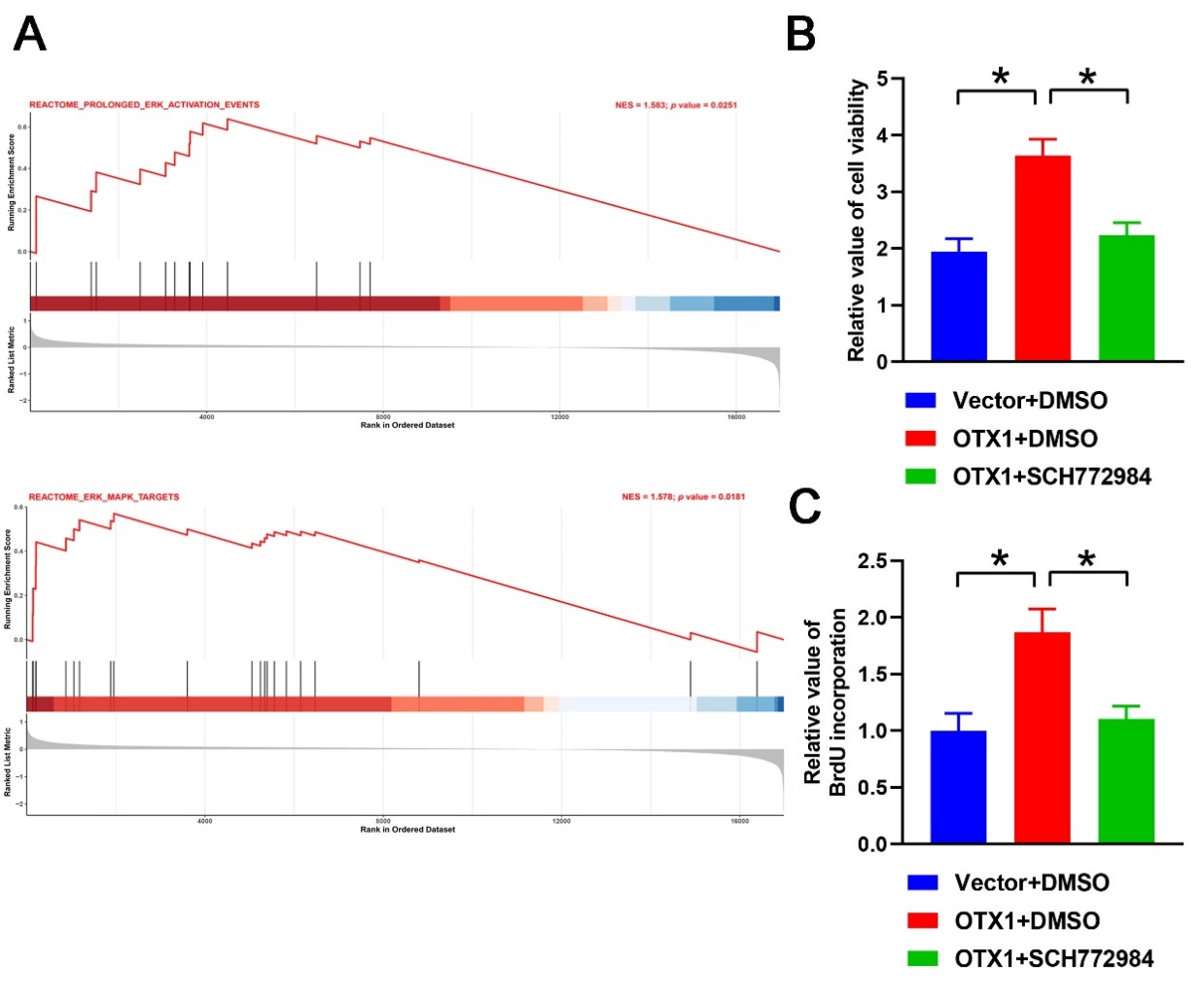


**Figure S6**

Involvement of the ERK signaling pathway in OTX1-mediated cellular proliferation in TNBC. A: Gene Set Enrichment Analysis (GSEA) revealed a positive correlation between genes implicated in ERK pathway activation and heightened OTX1 expression in TNBC (REACTOME_PROLONGED_ERK_ACTIVATION_EVENTS, NES = 1.583, p = 0.0251; REACTOME_ERK_MAPK_TARGETS, NES = 1.578, p = 0.0181). B and C: Enhanced cell viability (B) and BrdU incorporation (C) as a result of OTX1 overexpression are abrogated by ERK pathway inhibition.
